# On the Use of Chloral Hydrate in Lethargic Somnolency

**Published:** 1883-04

**Authors:** J. C. Burnett

**Affiliations:** London


### ON THE USE OF CHLORAL HYDRATE IN LETHARGIC SOMNOLENCY.

J. C. Burnett, M. D., London.

Those who have watched *old* chloral-eaters may have noticed that they slowly get lethargic, somnolent, and listless. Toward the end of the chapter of chronic chloralism there is a condition of fatty degeneration of a slow, lazy type, and the very mode of death seems peculiar. I have seen a case where the subject of chronic chloralism lay for days a-dying; she was for several days so that it was very difficult to determine whether she was dead or not.

Occasionally one comes across a remarkable case of somnolence, and then the narcotics are to be thought of by the therapeutist.

I will shortly relate two such cases from my own practice.

No. 1. A lady of about forty-five, stout, fresh-looking, and the mother of a family, was the subject of remark of her friends on account of her lethargy and sleepiness. Her weakness was such that even crossing the street was almost impossible; the weakness was peculiarly lethargic, a kind of listless heaviness. She was almost constantly asleep; she would get up in the morning after a good night's rest, and even while dressing she seemed compelled to sit down, and no sooner seated but she would fall asleep. This state of things went on for weeks and months, and her allopathic adviser did his best in vain. After she came under my care I tried first *Arnica*, and then *Opium*, with but indifferent success, when all at once I bethought me of the great similarity of the case before me to that of a confirmed old chloral-eater of my *clientèle.*

Chloral in a low dilution cured my patient, and she again became brisk, active, and wideawake.

No. 2. An elderly lady came under my care on April 21st, 1881, for lethargy, languor, and somnolence.

℞ Trit. 2x *Chloral Hydrat.* ℥ iv.

To take six grains in water every three hours.

May 7th. Under this date I find these notes in my case-book: "Feels a different creature; vastly improved; less lethargic, and decidedly less languid."

She then got the third decimal trituration in lieu of the second, and only two doses a day, and then needed no further treatment, as she subsequently informed me when calling with her husband.

Perhaps some of my colleagues have had similar experience of the use of the great sleep-giver to cure sleepiness.

It is a standing marvel to me how it is that Homœopathy does not carry conviction to people's minds.—*Homœopathic World.*

